# NSX-Net: A Neurolinguistic and Acoustic Multimodal Deep Learning Framework for Speech Disorder Classification

**DOI:** 10.3390/diagnostics16152377

**Published:** 2026-07-28

**Authors:** Adnan Nadeem, Mohammad Zubair Khan, Mehreen Sirshar, Usharani Thirunavukkarasu

**Affiliations:** 1 Faculty of Computer and Information Systems, Islamic University of Madinah, Medina 42351, Saudi Arabia; adnan.nadeem@iu.edu.sa; 2 King Salman Center for Disability Research, Riyadh 11614, Saudi Arabia; 3 Department of Software Engineering, Fatima Jinnah Women University, Rawalpindi 46000, Punjab, Pakistan; msirshar@gmail.com; 4 Department of Biomedical Engineering, Saveetha Engineering College, Chennai 602105, India; usharanithirunav@gmail.com

**Keywords:** speech disorder, diagnostics of speech disorder, multimodal speech disorder analysis, attention-based deep learning, disordered speech classification

## Abstract

**Background/Objectives**: Speech disorders caused by neurological, articulatory, cognitive, and behavioral impairments require accurate multimodal analysis frameworks capable of jointly understanding acoustic abnormalities and neurolinguistic inconsistencies for reliable computer-assisted speech disorder assessment. Recent multimodal deep learning approaches have utilized speech signals, linguistic transcripts, attention mechanisms, and contextual fusion strategies to improve disordered speech analysis and intelligent disorder prediction. However, existing methods frequently suffer from insufficient temporal synchronization, ineffective local–global feature learning, multimodal redundancy, poor interpretability, and reduced robustness under heterogeneous speech disorders. To address these challenges, **Methods**: The proposes NSX-Net (NeuroSpeech Explainable Network), an intelligent multimodal deep learning framework for speech disorder classification using Acoustic Speech Signal Data and Neurolinguistic Text/Linguistic Data. Initially, the Adaptive Speech Refinement Module (ASRM) performs noise removal, silence elimination, signal normalization, spectrogram generation, MFCC extraction, and transcript preprocessing to improve multimodal speech consistency. Subsequently, the Hierarchical Multimodal Feature Learning Unit (HMFLU) extracts discriminative feature representations through the Cross-Domain Representation Encoder (CDRE), Fine-Grained Speech Pattern Analyzer (FGSPA), and Global Sequential Dependency Learner (GSDL) for capturing both local articulation abnormalities and long-range semantic dependencies. Furthermore, the Dual-Path Attention Enhancement Block (DPAEB) emphasizes clinically important disordered speech regions using adaptive local–global attention mechanisms, while the Temporal Resolution Synchronization Module (TRSM) aligns rhythm-level and beat-level multimodal contextual structures to improve temporal consistency and suppress noise disturbances. **Results**: Experimental evaluation across six publicly available multimodal speech disorder datasets demonstrated that NSX-Net achieved superior performance, with 99.27% Accuracy, 99.11% Precision, 98.97% Recall, 99.02% F1-Score, and 99.41% AUC, significantly outperforming existing state-of-the-art frameworks. **Conclusions**: Finally, optimized multimodal contextual representations are forwarded into a Softmax classification layer for intelligent multiclass speech disorder prediction with improved interpretability and potential clinical applicability.

## 1. Introduction

Communication disorders include speech disorders, which affect an individual’s ability to produce meaningful speech. This is important because it will have an effect on their socialization, learning, emotions, and employment, among other areas. Speech articulation disorder, dysarthria, speech apraxia, stuttering, aphasia, and phonology disorders are some of the disorders that arise due to a variety of factors including neurological problems, development conditions, movement disorders, cognitive disorders, or voice tract abnormalities. Since early detection of any speech problems can improve the patient’s prognosis, speech disorder analysis has recently become an important field for health care professionals, neurologists, and linguists [1,2]. Human speech is made possible through complex neuromuscular activity among the brain, breathing apparatus, vocal cords, tongue, lips, and other articulators. Voice instability, breaks in the voice, uneven rhythm, articulation problems, and atypical speech pauses are some acoustic anomalies that may be brought about by any disruption in these systems. Traditional diagnostic techniques in the field usually depend on the subjective observation and listening of a speech–language therapist, often being subjective, time-intensive, and expert-dependent [3]. To facilitate objective diagnosis and real-time monitoring, speech disorder analysis technologies have gained prominence. Moreover, modern speech disorder diagnostics focus on neurolinguistic features like semantic consistency, lexical richness, grammatical consistency, and cognitive speech behavior, along with acoustic anomalies. Considering that speech abnormalities may affect both the voice and language simultaneously, the fusion of acoustic speech signals and neurolinguistic linguistic features yields more comprehensive insights into speech abnormalities [4,5]. This is why multimodal speech analysis has proved to be an effective way of improving computer-assisted speech assessment, personalized treatment planning, and clinical decision support in speech pathology systems.

A number of advanced computational algorithms and deep learning approaches have been devised to obtain discriminant linguistic and acoustic features from multimodal speech data for efficient evaluation of complex speech disorders. Before feature extraction, several preprocessing approaches such as noise reduction, elimination of silences, normalization of the signal, segmentations, and generation of spectrograms are applied for improving the reliability and quality of voice signals. Several aspects related to speech such as Mel Frequency Cepstral Coefficients (MFCCs), pitch, formants, jitter, shimmer, harmonics-to-noise ratio, speaking speed, and rhythms are extracted by acoustic processing approaches for identifying anomalies in speech associated with neurological and motor speech disorders [6,7]. Simultaneously, several neurolinguistic processing approaches are employed for evaluating text-based features like sentence structure, semantics, lexical diversity, context awareness, repeated word usage, and cognitive fluency. Although RNNs, LSTMs, and BiLSTMs have been found to be effective in modeling temporal dependencies between speech signals, CNNs have become prevalent in spatial feature extraction from spectrograms with the emergence of deep learning [8]. Transformers and models utilizing attention mechanisms have proven to be highly effective at modeling semantic relationships and long-term context relations in speech and linguistic data recently. Because attention mechanisms focus on clinically important speech regions by emphasizing the relevant temporal features, they increase the interpretability of the results. Additionally, multimodal methods integrate auditory and linguistic information in order to generate superior speech pathology diagnosis results by unifying the data in the feature space. To increase the reliability, robustness, and clinical viability of automatic speech disorder detection systems, cross-modal learning, hierarchical feature extraction, temporal alignment, and interpretable machine learning methods are increasingly being incorporated into state-of-the-art designs [9,10].

Various speech disorder analysis models based on machine learning and deep learning have emerged recently for detecting neurological and articulation impairments using speech signal analysis. Earlier systems were primarily based on handcrafted acoustic feature extraction, along with conventional classifiers such as SVM, RF, GMM, and HMM for detection of dysarthria, Parkinsonian speech, stuttering, and speech abnormalities associated with aphasia. However, later on, deep learning approaches based on CNN, LSTM, and hybrid CNN-LSTM models achieved better results by automatically deriving hierarchical acoustic representations from spectrogram and temporal voice signals [11,12]. Furthermore, various research works utilized deep neural networks with MFCC features for detection of speech disorders. Models based on Natural Language Processing (NLP) techniques for detecting cognitive deficits associated with neurodegenerative diseases and other language impairments using semantic and syntactic anomalies in voice transcription gained much more complexity after being studied in neurolinguistics. The recent multimodal models incorporate both verbal and audio signals to enhance the precision and context-understanding capabilities [13,14,15]. However, the current systems cannot effectively recognize the complicated relation between neurolinguistic processes and speech impairment; instead, they focus only on acoustics parameters and narrow linguistic details. In addition, the existing models lack robustness to the background noise and speech articulation variance, generalizability for different speech impairments, interpretability, and long-range temporal dependencies [16,17]. Moreover, certain approaches demonstrate computational redundancy due to inadequate multimodal synchronization algorithms and suboptimal feature extraction procedures. Additionally, accurate classification remains challenging due to the inherent clinical imbalance and highly diverse speech patterns in many speech disorder databases. The existing models are further constrained in their ability to identify minor articulation errors and unique features associated with specific speech disorders without incorporating adaptive attention mechanisms and context-based alignment methodologies [18,19,20]. In order to enhance the discriminative power of feature extraction, context-based learning, and overall classification performance in speech disorder detection tools, a viable, interpretable, and multimodal deep learning approach capable of analyzing both acoustic and neurolinguistic signals simultaneously is sorely required. This work introduces NSX-Net (NeuroSpeech Explainable Network), an advanced multilingual deep learning system that intelligently classifies speech disorders based on Neurolinguistic Text/Linguistic Data and Acoustic Speech Signal Data in order to circumvent these limitations. The proposed NSX-Net system effectively utilizes adaptive preprocessing, hierarchical multimodal learning, dual-path attention mechanism, synchronization of temporal resolution, and Softmax classification in order to detect different types of speech disorders.

To improve the overall scientific quality and reproducibility of the proposed framework, the manuscript has been comprehensively revised with enhanced methodological descriptions, consistent mathematical notation, standardized module terminology, improved figure and table captions, updated literature, and refined technical writing. These revisions improve the clarity, readability, and technical presentation of the proposed NSX-Net framework. Unlike conventional multimodal speech disorder classification frameworks that primarily rely on direct feature concatenation, single-stage attention mechanisms, or independent acoustic and linguistic processing, the proposed NSX-Net introduces a hierarchical multimodal learning strategy that jointly integrates Adaptive Speech Refinement (ASRM), Hierarchical Multimodal Feature Learning (HMFLU), Cross-Domain Representation Encoding (CDRE), Fine-Grained Speech Pattern Analysis (FGSPA), Global Sequential Dependency Learning (GSDL), Dual-Path Attention Enhancement (DPAEB), and Temporal Resolution Synchronization (TRSM). Each module is specifically designed to address limitations of existing approaches, including multimodal redundancy, insufficient cross-modal interaction, limited temporal dependency modeling, and inadequate feature discrimination. This unified architecture enables more robust multimodal feature representation and improved speech disorder classification across heterogeneous datasets.

## 2. Literature Survey

Algaraady et al. [21] adopted spectrogram and acoustic analysis to evaluate pharyngeal sound articulation in analyzing the neurolinguistic and acoustic dimensions of speech difficulties in the Arabic language. In this research, no automated categorization was done using a deep learning technique; however, there were significant inconsistencies observed in terms of pitch, jitter, shimmer, and voice. Drougkas et al. [22] proposed a multimodal machine learning system that would use linguistics and audio information to determine specific cues associated with language and voices for psychological problems. With their approach, the efficiency of psychological disorder analysis improved; however, contextual multimodal feature alignment was limited. Al-Shqeerat et al. [23] provided an AI-powered multimodal developmental disability recognition system with speech and behavior data along with the BioNeuroFusionNet classifier. While the proposed multimodal system required a high computational cost for multimodal synchronization, the model improved the accuracy of developmental disability recognition. Anuprabha et al. [24] proposed a multimodal dysarthria identification and severity evaluation approach based on speech and text features. While their system performed well in terms of predicting severity, subtle articulation issues could not be solved with fine-grained context extraction. Li et al. [25] proposed a multimodal model for the automated screening of children’s reading disorders using speech, language, and eye tracking information. While the method helped in improving the automation process, it was still not easy to capture semantic relations from afar.

Zhao et al. [26] designed a triplet multimodal transfer learning algorithm for the screening of speech problems in PD patients based on acoustic speech signals. Though the system mostly focused on analyzing speech signals rather than neurolinguistics, their transfer learning algorithm improved classification accuracy. Wu et al. [27] proposed the multimodal dual-CNN recursion model (MDCNN) for identifying fake news using speech emotion recognition from text and audio. While temporal consistency was not perfectly considered, the proposed model effectively extracted speech emotions. Poor et al. [28] used speech, language, and image information to develop a multimodal cross-transformer architecture for predicting moderate cognitive impairment. Though the cross-transformer structure improved the performance of cognitive diseases classification, multimodal learning increased computational expenses. Ghosh et al. [29] utilized a transformer-based hybrid multimodal framework for recognizing psychiatric diseases based on different modes of representation. Though interpretability and noise robustness were still limited, their approach improved emotion disorder detection. Ksibi et al. [30] built a multimodal Siamese framework for the diagnosis of dementia from female vocal information. Even though the method proved efficient in identifying dementia, unbalanced multimodal feature distribution posed challenges in its implementation.

Ortiz-Perez et al. [31] introduced CogniAlign as a word-level multimodal speech alignment framework using gated cross-attention that could be used for the detection of Alzheimer’s disease. While semantic understanding was better through their alignment method, local speech irregularities remained insufficiently modeled. Xing et al. [32] proposed an adaptive multi-graph neural network along with multimodal feature fusion learning for the diagnosis of severe depression. Although issues with temporal speech were not entirely addressed, the multimodal learning process benefited significantly from graph-based fusion. Zhang et al. [33] introduced a multimodal system that detects depression through texts, audio, and videos. While their use of the multimodal system improved their accuracy, there was a need to introduce better approaches to attention in order to prioritize features. Jia et al. [34] proposed a multimodal system for detecting depression that used the attention graph convolution and transformer networks. While there was some improvement in reducing feature duplications, the approach was able to identify emotions well through different modes. KANAN et al. [35] proposed a multimodal major depressive illness identification framework based on EEG and auditory information. While the system needed a high number of dimensions to capture emotions, their multimodal system helped detect emotional disorders well.

Yang et al. [36] proposed the MMPF, which stands for multimodal purification fusion framework, for automated depression detection based on multiple modes. While noise filtering was optimized through the process of purification fusion, local contextual feature extraction remained inadequate. Yousufi et al. [37] developed a multimodal fusion framework for the detection of depression disorders with EEG and spectrogram modality features, leveraging a modified DenseNet121 model. Though the approach raised multimodal classification precision, further improvements were required regarding interpretability. Kapil et al. [38] introduced a transformer-based multitask learning framework for multimodal hate speech detection with textual and visual modalities. Despite limited cross-modal temporal dependency learning, the multitask transformer improved semantic understanding. Chhabra et al. [39] introduced MHS-STMA, a scalable multilevel attention-based transformer framework for multimodal hate speech detection. Even though their multilayer attention method increased computational complexity substantially, it had a positive effect on contextual representation learning. Tao et al. [40] introduced DepMSTAT, a multimodal spatiotemporal attention-based transformer framework for depression detection using heterogeneous behavioral data. While spatiotemporal attention helped improve temporal emotion analysis, fine-grained multimodal synchronization was still hard to achieve.

Elena et al. [41] proposed DEPART (Depression and Parkinson’s Recognition Technique), an integrated multitask model for recognizing depression and Parkinson’s disease using wild-video data, which encompasses CLIP-based visual encoding, body region extraction, Transformer-based temporal modeling, and prototype-aware gated fusion classification. The model adapts gradient-based attention maps to enhance interpretability by highlighting task-specific visual regions that contribute to the prediction process. Pan et al. [42] introduced the Two-Step Attention-based Feature Combination Cross-Attention (TSAC-ATT) framework for speech-based dementia detection, leveraging self-supervised learning (SSL) models to extract robust acoustic and linguistic representations. The proposed cross-attention feature fusion strategy effectively combines contextual features from SSL models, achieving superior dementia detection performance compared to conventional feature combination methods. Liu et al. [43] introduced a wearable, wireless, flexible skin-attached acoustic sensor (SAAS) that captures vocal organ vibrations and skin movements to enable speech recognition and human–machine interaction in harsh acoustic environments. By integrating machine learning with wearable acoustic sensing, the system enhances robust voice recognition performance even under significant environmental noise. The proposed NSX-Net framework introduces an advanced explainable multimodal deep learning architecture for intelligent speech disorder classification using synchronized acoustic and neurolinguistic feature learning. To accomplish this, we:

- Proposed a novel NSX-Net, an explainable multimodal deep learning architecture that jointly exploits acoustic speech signals and neurolinguistic textual representations for intelligent speech disorder classification.
- Developed the Hierarchical Multimodal Feature Learning Unit (HMFLU), consisting of the Cross-Domain Representation Encoder (CDRE), Fine-Grained Speech Pattern Analyzer (FGSPA), and Global Sequential Dependency Learner (GSDL), to effectively capture complementary local and global multimodal representations.
- Introduced the Dual-Path Attention Enhancement Block (DPAEB) and Temporal Resolution Synchronization Module (TRSM) to enhance multimodal feature refinement, temporal consistency, contextual alignment, and interpretability.
- Conducted comprehensive experimental evaluations on six publicly available multimodal speech disorder datasets using multiple performance metrics, demonstrating superior classification performance compared with existing state-of-the-art approaches.

Recent speech foundation models and transformer-based architectures have significantly improved multimodal speech representation learning through self-supervised pretraining and large-scale contextual modeling. Similarly, explainable multimodal clinical AI frameworks have demonstrated improved transparency in neurological disorder assessment. However, most existing approaches primarily focus on either acoustic representation learning, linguistic modeling, or transformer-based contextual analysis individually, while limited attention has been given to jointly optimizing hierarchical multimodal feature extraction, cross-domain representation learning, adaptive attention enhancement, and temporal synchronization within a unified framework. Unlike those methods, the proposed NSX-Net integrates these complementary modules to improve multimodal feature interaction, robustness, interpretability, and cross-dataset speech disorder classification performance.

## 3. NSX-Net Framework

The proposed NSX-Net (NeuroSpeech Explainable Network) is a multimodal deep learning framework introduced for intelligent classification of speech disorders utilizing Acoustic Speech Signal Data and Neurolinguistic Text/Linguistic Data within a unified feature learning architecture. The framework combines adaptive preprocessing, hierarchical multimodal feature extraction, attention-guided feature learning, temporal synchronization, and Softmax-based classification to precisely recognize disordered speech abnormalities with enhanced interpretability, robustness, and clinical consistency.

A. Multimodal Speech Acquisition and Adaptive Preprocessing

The proposed NSX-Net (NeuroSpeech Explainable Network) begins by acquiring two heterogeneous input modalities, namely, Acoustic Speech Signal Data and Neurolinguistic Text/Linguistic Data, for comprehensive multimodal speech disorder analysis. Let the acoustic speech signal be denoted as S(t), where t∈[1,T] represents the temporal speech sequence, and T is the total signal duration. Similarly, the neurolinguistic textual sequence is denoted as $L=\left\{w_{1},w_{2},w_{3},\ldots ,w_{n}\right\}$, where $w_{i}$ represents the i-th linguistic token, and n is the total number of transcript words. These multimodal inputs are performed through ASRM to normalize the speech amplitude, eliminate noise, generate spectrograms, remove silence regions, and prepare contextual linguistic demonstrations for downstream multimodal learning. Initially, acoustic noise removal is processed utilizing spectral filtering to suppress unnecessary background disturbances from disordered speech recordings, where the denoised speech signal $S_{d}\left(t\right)$ is acquired as:

(1) $$S_{d}\left(t\right)=S\left(t\right)-N\left(t\right)$$

where S(t) is denoted as the original speech signal, N(t) is the evaluated background noise component, and $S_{d}\left(t\right)$ is the denoised speech signal. After denoising, signal normalization is adapted to handle the amplitude consistency across heterogenous speech recordings. The normalized speech signal $S_{n}\left(t\right)$ is generated as:

(2) $$S_{n}\left(t\right)=\frac{S_{d}\left(t\right)-\mu }{\sigma }$$

where μ represents speech signal mean amplitude, σ is the speech signal standard deviation, and $S_{n}\left(t\right)$ is the standardized speech signal. To remove inappropriate pauses and silent intervals, voice activity detection is processed through the adaptive threshold θ. The silence-eliminated signal $S_{r}\left(t\right)$ is:

(3) $$S_{r}\left(t\right)=\{\begin{array}{cc} S_{n}\left(t\right), & \left|S_{n}\left(t\right)\right|>\theta \\ 0, & otherwise \end{array}$$

where θ is the threshold of silence detection, and $S_{r}\left(t\right)$ is the enhanced speech signal after silence elimination. The enhanced speech signal is then converted into a spectrogram demonstration utilizing Short-Time Fourier Transform (STFT) to capture temporal frequency characteristics coordinated with articulation abnormalities. The spectrogram X(f,τ) is expressed as:

(4) $$X\left(f,\tau \right)=\sum_{t=-\infty }^{\infty }S_{r}\left(t\right)w\left(t-\tau \right)e^{-j2\pi ft}$$

Here, X(f,τ) denotes the time–frequency spectrogram representation, w(t−τ) is the windowing function, f is the frequency component, τ is the temporal frame index, and j is the imaginary unit. Then, Mel-Frequency Cepstral Coefficients (MFCCs) are extracted to acquire a compact acoustic demonstration of vocal characteristics and articulation patterns. The MFCC feature vector $M_{k}$ is generated as:

(5) $$M_{k}=\sum_{m-1}^{M}\log\left(Y_{m}\right)\cos\left[\frac{\pi k}{M}(m-0.5)\right]$$

where $M_{k}$ is the k-th MFCC coefficient, $Y_{m}$ is the Mel-scaled spectral energy, M is the total Mel filters banks, and k is the cepstral coefficient index. For neurolinguistic preprocessing, the textual sequence is transformed and tokenized into feature embeddings for semantic analysis. The linguistic embedding demonstration $E_{i}$ for each token is:

(6) $$E_{i}=Embed\left(w_{i}\right)$$

Here, $E_{i}$ is the feature embedding token vector $w_{i}$, Embed(·) represents the embedding transformation operation, and $w_{i}$ is the input linguistic token. Finally, the preprocessed acoustic representation $A_{f}$ and linguistic representation $L_{f}$ are integrated to form the enhanced multimodal input space:

(7) $$F_{multi}=A_{f}⊕L_{f}$$

where ⊕ denotes the multimodal concatenation operation, and $F_{multi}$ is the final multimodal reinforced feature space. Thus, the ASRM efficiently standardizes heterogeneous multimodal speech inputs, suppresses pathological noise instabilities, and enhances the feature consistency for robust downstream speech disorder analysis within NSX-Net.

B. Hierarchical Multimodal Feature Extraction and Contextual Representation Learning

**Figure 1:**
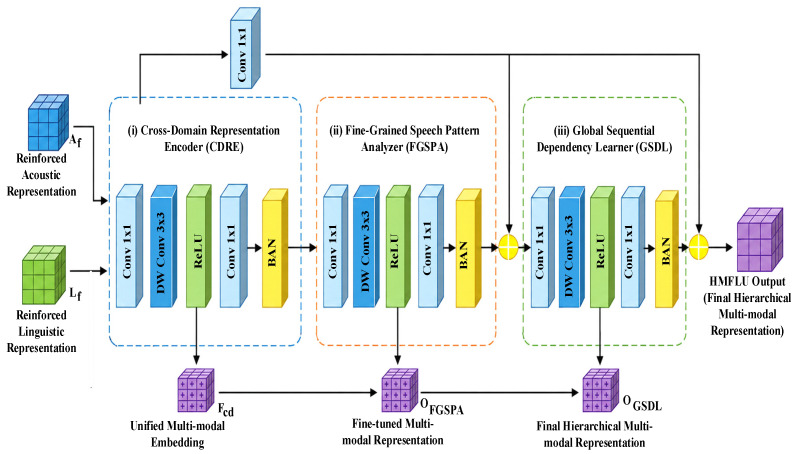
HMFLU module hierarchically integrates cross-domain multimodal encoding, fine-grained articulation analysis, and global sequential feature learning to generate highly discriminative multimodal speech representations for accurate speech disorder classification.

**Figure 2:**
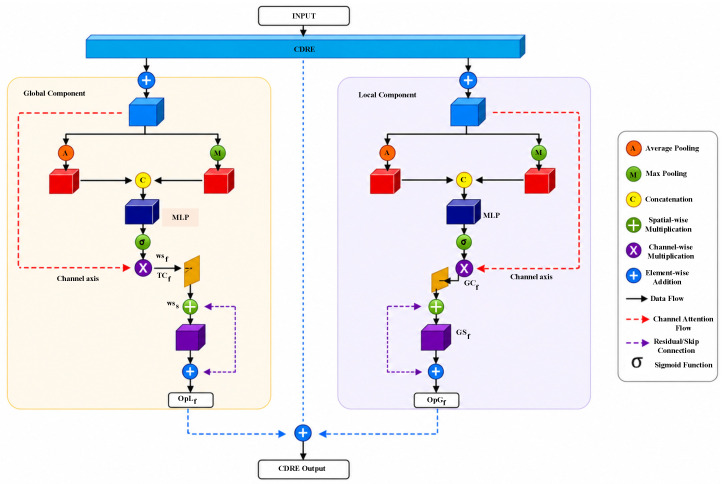
CDRE module jointly learns global semantic dependencies and local fine-grained disordered speech representations through adaptive channel–spatial attention mechanisms to generate enriched multimodal contextual features for robust speech disorder analysis.

After preprocessing, the reinforced acoustic and neurolinguistic representations are passed into HMFLU (Hierarchical Multimodal Feature Learning Unit) for efficient multimodal feature extraction and feature learning depicts in Figure 1. This module plays a vital role in detecting both fine-tuned articulation abnormalities and long-range semantic speech dependencies for precise speech disorder classification. Assume the reinforced acoustic speech demonstration can be represented as $A\in R^{h\times w\times c}$ and the linguistic context represented as $L\in R^{h\times w\times c}$, where h,w and c are the height, width and channel dimension, respectively. HMFLU mainly comprises three major components: Cross-Domain Representation Encoder (CDRE), Fine-Grained Speech Pattern Analyzer (FGSPA), and Global Sequential Dependency Learner (GSDL) as shown in Figure 2.

(i) Cross-Domain Representation Encoder (CDRE)

CDRE combines and arranges multimodal acoustic and linguistic features into a unified feature embedding space, thereby enhancing the interconnection between speech rhythm characteristics and semantic linguistic representations. This process effectively minimizes the modality gap among acoustic speech signals and neurolinguistic textual features while enhancing the discriminative learning competence for disordered speech understanding. The multimodal contextual fusion process is defined as:

(8) $$F_{cd}=CDRE\left(A_{f},L_{f}\right)$$

where $A_{f}$ and $L_{f}$ are denoted as the refined acoustic feature and linguistic contextual representation, CDRE(·) is the cross-domain multimodal encoding function, and $F_{cd}$ is the unified multimodal feature embedding. Initially, shallow multimodal representations are extracted self-sufficiently from acoustic and linguistic modalities expressed as:

(9) $$\theta_{a}={Conv}_{1\times 1}\left(A_{f}\right)$$

(10) $$\theta_{l}={Conv}_{1\times 1}\left(L_{f}\right)$$

where $\theta_{a}$ and $\theta_{l}$ represent the shallow embedding of acoustic and linguistic features. ${Conv}_{1\times 1}$ is the channel minimization convolution operation. Moreover, bottleneck residual learning is applied using a stacked 3 × 3 convolution operation to facilitate multimodal feature learning and accelerate convergence.

(ii) Fine-Grained Speech Pattern Analyzer (FGSPA)

FGSPA captures subtle articulation deviations, pause irregularities, pronunciation inconsistencies, and micro-level pitch variations necessary for disordered speech analysis. Initially, local multimodal features are extracted self-sufficiently from acoustic and linguistic embeddings:

(11) $$L_{a}\in R^{h\times w\times c}$$

(12) $$L_{l}\in R^{h\times w\times c}$$

where $L_{a},L_{l}$ denotes the local contextual features of acoustic and linguistic inputs. To enhance feature discrimination, channel attention weights are determined through global max pooling and average pooling operations followed through multilayer perceptron learning:

(13) $$W_{a}=\sigma \left(MLP\left(MPOOL\left(L_{a}\right)+APOOL\left(L_{a}\right)\right)\right)$$

(14) $$W_{l}=\sigma \left(MLP\left(MPOOL\left(L_{l}\right)+APOOL\left(L_{l}\right)\right)\right)$$

where $W_{a},W_{l}$ are the channel attention weights of acoustic and linguistic inputs, MPOOL(·) is the global max pooling, and APOOL(·) is the global average pooling (GAP), MLP(·) is the multilayer perceptron, and σ is the sigmoid activation function. The computed attention weights are multiplied with local multimodal features to highlight clinically significant articulation structures:

(15) $$T_{a}=W_{a}⊗L_{a}$$

(16) $$T_{l}=W_{l}⊗L_{l}$$

where $T_{a},T_{l}$ are the channel-improved acoustic and linguistic features, and ⊗ denotes the channel-wise multiplication operation. Next, spatial attention mechanisms are used to dynamically recognize related disordered speech regions:

(17) $$S_{a}=\sigma \left({Conv}_{1\times 1}\left(ReLU\left({Conv}_{1\times 1}\left(T_{a}\right)\right)\right)\right)$$

(18) $$S_{l}=\sigma \left({Conv}_{1\times 1}\left(ReLU\left({Conv}_{1\times 1}\left(T_{l}\right)\right)\right)\right)$$

where $S_{a}$ and $S_{l}$ represent the spatial attention map of acoustic and linguistic inputs. Finally, spatially reinforced multimodal representations are obtained as:

(19) $$P_{a}=S_{a}⨀T_{a}$$

(20) $$P_{l}=S_{l}⨀T_{l}$$

Here, $P_{a},P_{l}$ are the refined representation of acoustic disordered speech and linguistic contextual features, and ⨀ denotes the spatial-wise multiplication operation. The final fine-tuned contextual result is computed by residual feature fusion:

(21) $$O_{FGSPA}=P_{a}+P_{l}$$

where $O_{FGSPA}$ represents the final fine-tuned multimodal speech representation.

(iii) Global Sequential Dependency Learner (GSDL)

GSDL models long-range temporal speech dependencies and eliminates redundant contextual information for enhanced global disordered speech understanding. Unlike FGSPA, which concentrates on abnormalities of local articulation, GSDL focuses on capturing large-scale semantic continuity and rhythm structures relationships from multimodal speech demonstrations. Initially, global feature representations are extracted from the embedding space of unified multimodal data:

(22) $$G_{a}\in R^{h\times w\times c}$$

(23) $$G_{l}\in R^{h\times w\times c}$$

where $G_{a},G_{l}$ represent the global contextual representation of acoustic and linguistic inputs. GSDL adapts contextual filtering at a high level to capture long-range temporal dependencies:

(24) $$H_{a}=HighPass\left(G_{a}\right)$$

(25) $$H_{a}=HighPass\left(G_{l}\right)$$

The optimization of channel and spatial attention are subsequently adapted to global feature representations:

(26) $$C_{a}=\sigma \left(MLP\left(H_{a}\right)\right)$$

(27) $$C_{l}=\sigma \left(MLP\left(H_{l}\right)\right)$$

(28) $$R_{a}=C_{a}⨀H_{a}$$

(29) $$R_{l}=C_{l}⨀H_{l}$$

where $C_{a},C_{l}$ are global contextual attention weights, and $R_{a},R_{l}$ indicate the global representations of refined multimodal contextual information. Finally, residual contextual fusion integrates the original multimodal embeddings with globally reinforced semantic dependencies:

(30) $$O_{GSDL}=F_{cd}+R_{a}+R_{l}$$

where $O_{GSDL}$ denotes the final global sequential contextual representation, and $F_{cd}$ is the unified multimodal feature embedding obtained from CDRE. Thus, the HMFLU efficiently extracts both local articulation abnormalities and global semantic dependencies, permitting extremely discriminative multimodal speech representations for accurate downstream speech disorder classification within NSX-Net.

C. Attention Enhancement and Multimodal Synchronization

After feature extraction, the multimodal contextual features computed through HMFLU are passed into DPAEB and TRSM to enhance contextual reliability, multimodal synchronization and disordered speech localization. Let the local contextual demonstration extracted from FGSPA be denoted as $F_{L}$ and the global contextual demonstration computed by GSDL be denoted as $F_{G}$. These representations are reinforced utilizing adaptive attention understanding and temporal synchronization strategies to highlight clinically significant articulation abnormalities and semantic irregularities while suppressing inappropriate noise features.

(i) DPAEB

**Figure 3:**
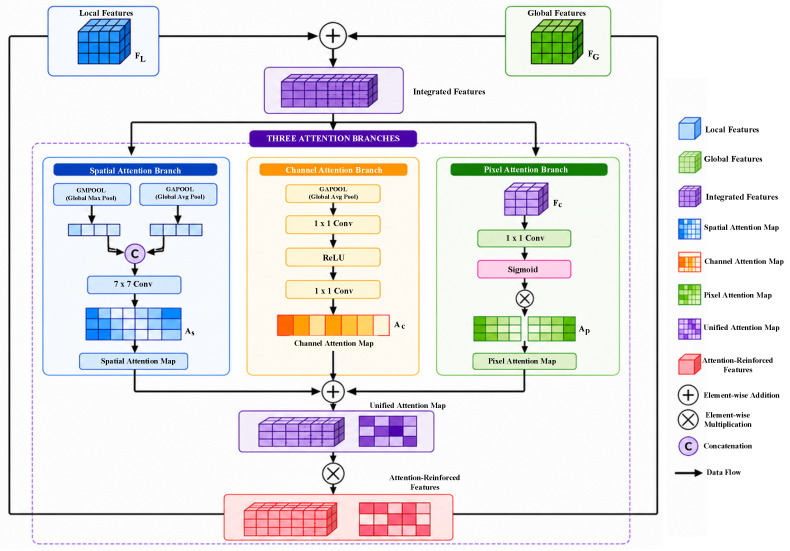
DPAEB module enhances multimodal disordered speech representations by jointly integrating spatial, channel, and pixel attention mechanisms to emphasize clinically significant articulation regions and contextual semantic abnormalities for improved interpretability and classification performance.

As illustrated in Figure 3, DPAEB enhances multimodal feature interpretability through integrating local and global attention mechanisms. Initially, contextual features at the local and global levels are fused as:

(31) $$F_{C}=F_{L}+F_{G}$$

where $F_{L},F_{G}$ are denoted as multimodal contextual features at the local and global levels, and $F_{C}$ is the integrated multimodal representation. The DPAEB consists of three attention branches: Spatial attention branch, Channel attention branch, and Pixel attention branch.

Spatial Attention Branch: The spatial branch detects significant disordered speech regions utilizing global pooling functions:

(32) $$A_{S}={Conv}_{7\times 7}\left[GMPOOL\left(F_{C}\right),GAPOOL\left(F_{C}\right)\right]$$

where $A_{S}$ is the spatial attention map, GMPOOL denotes global max pooling, and GAPOOL is global average pooling. This branch supports the model to target neurologically affected articulation regions.

Channel Attention Branch: The channel branch determines significant multimodal channel dependencies:

(33) $$A_{C}={Conv}_{1\times 1}\left(ReLU\left({Conv}_{1\times 1}\left(GAPOOL\left(F_{C}\right)\right)\right)\right)$$

where $A_{C}$ is the channel attention map. This function highlights clinically related speech features.

Pixel Attention Branch: The pixel branch captures fine-tuned articulation inconsistencies of abnormalities and pronunciation:

(34) $$A_{P}={Conv}_{1\times 1}\left(SIGMOID\left(F_{C}\right)\right)$$

where $A_{P}$ represents the pixel-level attention map. Subsequently, in unified attention learning, the results from the overall attention branches are fused as:

(35) $$A_{F}=A_{S}+A_{C}+A_{P}$$

where $A_{F}$ denotes the final unified attention map. The representation of refined multimodal contextual information is then computed as:

(36) $$F_{A}=F_{C}⊗A_{F}$$

where $F_{A}$ is the attention-reinforced multimodal representation, and ⊗ is the element-wise multiplication. Thus, DPAEB enhances disordered speech localization and multimodal contextual understanding.

(ii) TRSM

**Figure 4:**
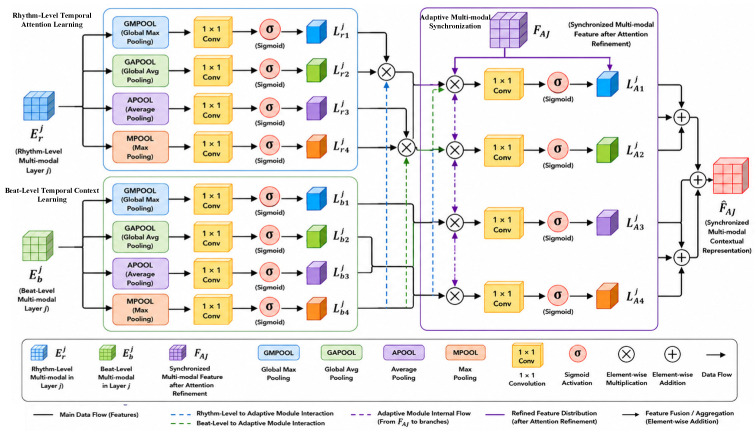
TRSM module adaptively synchronizes rhythm-level and beat-level multimodal feature representations through temporal attention learning and multimodal alignment mechanisms to enhance temporal consistency, contextual reliability, and disordered speech understanding.

After attention refinement, the improved multimodal features are passed into TRSM for adaptive multimodal temporal alignment and contextual synchronization. As illustrated in Figure 4, TRSM combines low-resolution rhythm-level structures with high-resolution beat-level multimodal features to enhance temporal reliability and suppress multimodal noise instabilities under heterogeneous speech disorders. Let the rhythm-level contextual representation and beat-level contextual demonstration be represented as $E_{r}^{j},E_{b}^{j}$, which are representations of the rhythm-level multimodal in layer j and the beat-level multimodal contextual in layer j.

Rhythm-Level Temporal Attention Learning

Initially, several pooling operations are used to capture temporal semantic dependencies from speech structures at the rhythm level:

(37) $$L_{r1}^{j}=SIGMOID\left(Conv\left(GMPOOL\left(E_{r}^{j}\right)\right)\right)$$

(38) $$L_{r2}^{j}=SIGMOID\left(Conv\left(GAPOOL\left(E_{r}^{j}\right)\right)\right)$$

(39) $$L_{r3}^{j}=SIGMOID\left(Conv\left(APPOL\left(E_{r}^{j}\right)\right)\right)$$

(40) $$L_{r4}^{j}=SIGMOID\left(Conv\left(MPOOL\left(E_{r}^{j}\right)\right)\right)$$

where $L_{r1}^{j},L_{r2}^{j},L_{r3}^{j},L_{r4}^{j}$ are denoted as rhythm-level temporal attention maps, APOOL(⋅) as average pooling, and MPOOL(⋅) as max pooling. These operations enhance the contextual understanding of speech rhythm articulation and indiscretions timing disturbances.

Beat-Level Temporal Context Learning: Similarly, feature representations at the beat level are enhanced utilizing temporal contextual attention learning, defined as:

(41) $$L_{b1}^{j}=SIGMOID\left(Conv\left(GMPOOL\left(E_{b}^{j}\right)\right)\right)$$

(42) $$L_{b2}^{j}=SIGMOID\left(Conv\left(GAPOOL\left(E_{b}^{j}\right)\right)\right)$$

(43) $$L_{b3}^{j}=SIGMOID\left(Conv\left(APPOL\left(E_{b}^{j}\right)\right)\right)$$

(44) $$L_{b4}^{j}=SIGMOID\left(Conv\left(MPOOL\left(E_{b}^{j}\right)\right)\right)$$

Here, $L_{b1}^{j},L_{b2}^{j},L_{b3}^{j},L_{b4}^{j}$ are denoted as beat-level contextual attention maps. These strategies improve the synchronization among articulation structures in the short term and temporal speech dependencies in the long range.

Adaptive Multimodal Synchronization: The enhanced attention representations are combined with the synchronized representation of the multimodal contextual feature $F_{AJ}$:

(45) $$L_{A1}^{j}=SIGMOID\left(Conv\left(GMPOOL\left(E_{A}^{j}\right)\right)\right)⊗F_{AJ}$$

(46) $$L_{A2}^{j}=SIGMOID\left(Conv\left(GAPOOL\left(E_{A}^{j}\right)\right)\right)⊗F_{AJ}$$

(47) $$L_{A3}^{j}=SIGMOID\left(Conv\left(APPOL\left(E_{A}^{j}\right)\right)\right)⊗F_{AJ}$$

(48) $$L_{A4}^{j}=SIGMOID\left(Conv\left(MPOOL\left(E_{A}^{j}\right)\right)\right)⊗F_{AJ}$$

where $L_{A1}^{j},L_{A2}^{j},L_{A3}^{j},L_{A4}^{j}$ represent the synchronized outcomes of multimodal temporal attention. Finally, the synchronized contextual demonstration computed through TRSM is acquired as:

(49) $${\hat{F}}_{AJ}=L_{A1}^{j}+L_{A2}^{j}+L_{A3}^{j}+L_{A4}^{j}$$

where ${\hat{F}}_{AJ}$ is the final contextual representation of the synchronized multimodal. Consequently, the TRSM efficiently arranges rhythm-level and beat-level speech representations, enhances multimodal temporal reliability, suppresses pathological noise disturbances, and reinforces contextual feature superiority for accurate downstream speech disorder classification within NSX-Net.

D. Speech Disorder Classification and Decision Optimization

In the final phase of the proposed NSX-Net, the synchronized multimodal feature representations computed through DPAEB and TRSM modules are passed into the Softmax Classification Layer for intelligent speech disorder prediction. This phase jointly examines acoustic irregularities, articulation distortions, semantic discrepancies, temporal rhythm unconventionalities, and neurological speech behavior fluctuations to precisely classify several speech disorders. The optimized multimodal feature representation attained from the earlier phase is denoted as:

(50) $$F_{final}={\hat{F}}_{AJ}$$

Initially, multimodal feature representations are converted into class prediction logits utilizing a fully connected classification layer:

(51) $$Z=W_{c}F_{final}+b_{c}$$

where $W_{c}$ is the weight matrix of trainable classification, and $b_{c}$ is a bias vector. Moreover, the Softmax activation function transforms the result logits into a distribution of probability for multiclass speech disorder prediction:

(52) $$P\left(Y_{i}\right)=\frac{e^{Z_{i}}}{\sum_{k=1}^{N}e^{Z_{k}}}$$

where $P\left(Y_{i}\right)$ denotes the probability of the i-th speech disorder class, $Z_{i}$ is the predicted score for class i, N is the total number of speech disorder categories, and e is the exponential function. Finally, the predicted speech disorder class is attained utilizing maximum probability selection:

(53) $$\hat{y}=\arg\max\left(P\left(Y_{i}\right)\right)$$

where $\hat{y}$ represents the final predicted speech disorder class, and argmax(⋅) is the index of the maximum probability category. Thus, the Softmax classification phase effectively performs robust multimodal speech disorder prediction by jointly utilizing articulatory, acoustic, linguistic, and temporal feature representations learned by NSX-Net.

## 4. Experimental Analysis

The efficiency of the proposed NSX-Net model for classifying speech disorders using multimodal speech signals and neurolinguistic text information in diverse cases of speech pathology is investigated in this part. The aim of comparison is to prove the efficiency of the NSX-Net approach regarding context-based learning, multimodal synchronization, classification accuracy, explainability, and model robustness in contrast to the other latest state-of-the-art models.

A. Dataset Description

TORGO Dataset: The TORGO speech corpus of individuals with dysarthria was created and made freely available for analysis of motor speech disorders caused by neurogenic diseases like cerebral palsy and ALS. Over 8000 speech files were obtained from patients with dysarthria and healthy controls, and they include individual words, short phrases, sentences, and spontaneously produced speech. The database includes multimodal information that enables acoustic and neurolinguistic analysis, e.g., transcribed language, articulatory kinematics data, and acoustic speech signals. The main emphasis of the data is on classes of speech disorders associated with fluctuations in the intensity of dysarthria. Dysarthria patients tend to have a lack of speech clarity, slurring of speech, rhythm disturbance, and unstable articulation. The dataset TORGO is best suited for evaluating the performance of multimodal deep learning approaches, such as NSX-Net, since it consists of highly diverse patterns of disordered speech and accompanying texts.

UA-Speech Dataset: UA-Speech is a disordered speech database that was developed particularly to serve as an experimental resource for studies on the speech intelligibility of dysarthria and automatic speech recognition. It comprises nearly 15 h of audio data from a set of 19 dysarthric subjects and 13 normal controls. These comprise numerical data, single words, uncommon words, alphabetic characters, and sentences. High-quality audio samples alongside their corresponding transcripts are provided, thus facilitating the simultaneous acquisition of acoustic and linguistic information. The two primary classes present within the dataset are dysarthria speech and normal speech. Furthermore, there are varying degrees of the extent to which the dysarthric speech recordings demonstrate difficulties ranging from mild to severe articulation issues. It serves as an ideal source for validating the effectiveness of multimodal models of speech disorder classification, as it exhibits highly distorted pronunciation patterns and impaired speech intelligibility.

AphasiaBank Dataset: The AphasiaBank dataset consists of an extensive multimodal speech and language impairment database developed to study cognitive linguistic disorders, as well as communication problems related to aphasia. Speech samples collected through the conversation tasks, storytelling, and description tasks of thousands of healthy neurological subjects and aphasic subjects were recorded. As a neurolinguistic research resource, AphasiaBank provides multimodal speech and language data including audio speech signals, text transcriptions, semantics, syntax, discourse features, and errors in speech and language. Due to the fact that it encompasses multiple forms of aphasia, such as Broca’s aphasia, Wernicke’s aphasia, anomia, and conduction aphasia, the dataset permits the analysis of semantic degradation and production issues in detail. The AphasiaBank corpus is exceptionally valuable in the assessment of contextual speech comprehension, semantic coherence learning, and multilingual cognitive speech pathology within the NSX-Net model.

**Figure 5:**
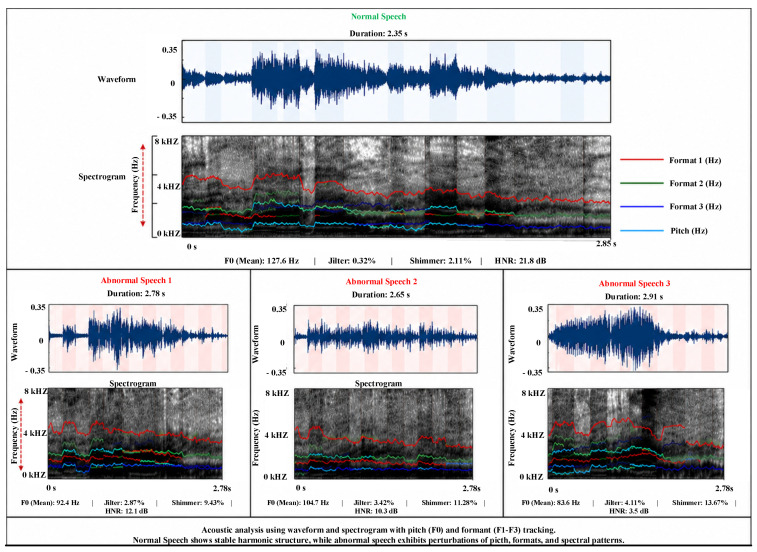
Demonstration that abnormal speech signals exhibit unstable pitch variations, increased jitter and shimmer values, reduced harmonic-to-noise ratio (HNR), and distorted spectrotemporal patterns compared with stable harmonic structures observed in normal speech.

Parkinson Speech Dataset: Neuromotor and voice degradation in Parkinsonian speech are often evaluated by the use of the Parkinson Speech Dataset. More than 5000 audio samples taken under standardized conditions of both patients suffering from Parkinson’s disease and normal subjects constitute the dataset. Voice acoustic features like jitter, shimmer, pitch variability, harmonics-to-noise ratio, vocal tremor, and frequency variation related to Parkinsonian speech are the predominant characteristics of this dataset. Although there are other expanded forms of the dataset, which contain severity-based progression information, the dataset usually involves binary classification classes representing either Parkinson’s disease or healthy control speech conditions. These pathological acoustic features in the dataset play an important role in measuring the accuracy of temporal speech analysis and speech abnormality identification through NSX-Net, owing to the high degree of vocal impairment caused by Parkinson’s disease. Figure 5 illustrates comparative acoustic waveform and spectrogram analysis between normal speech and abnormal disordered speech samples using pitch (F0) and formant (F1–F3) tracking.

DementiaBank Dataset: DementiaBank is an example of a multimodal speech language disorders database compiled for investigation of linguistics and spontaneous speech in relation to analyzing dementia and Alzheimer’s disease. Hundreds of hours of speech audio recordings from patients with dementia and normal older individuals performing cognitive tasks such as visual descriptions and memory tests are included in this database. To help with neurolinguistic evaluation, it includes synchronized audio files, transcriptions, semantics tagging, pause information, repeated word counts, and discourse-level linguistic differences. Although Alzheimer’s disease severity levels are mentioned in some subsets in the database, there are only two fundamental levels that can be classified as either cognitively normal speech or disordered speech caused by dementia. The DementiaBank dataset is highly helpful when confirming multimodal contextual dependency learning and cognitive speech disorder detection for NSX-Net because dementia significantly influences speech semantic, contextual, lexical, and temporal organization.

DAIC-WOZ Dataset: One widely used dataset for detecting psychological speech disorders, emotional distress, and depression is DAIC-WOZ (Distress Analysis Interview Corpus-Wizard of Oz). Around 189 sessions of clinical interviews with recorded audio clips, transcript texts, behavioral interaction data, and annotated emotional information collected through human–computer conversations are available in this dataset. It provides multimodal inputs that facilitate emotional as well as neurolinguistic analysis of speech, which include speech acoustic features, dialogue transcripts, facial expressions, and psychological annotations. Although more rating scores about depression severity have been included to make the analysis more accurate, the majority of the data contains only two categories representing either depressed people or non-depressed people. The main reason why the DAIC-WOZ dataset is useful in efficiently conducting experiments on the proposed NSX-Net model is that it contains synchronized multimodal information about emotion and language.

B. Experimental Setup

To design the proposed NSX-Net architecture for feature learning based on contextual cues and multimodal classification of the identified speech disorders, Python 3.11 was utilized together with the PyTorch deep learning library. In turn, for neurolinguistic analysis of transcripts, such tools as the HuggingFace Transformers API and NLTK library were employed, whereas LibROSA and Praat were used for performing several preprocessing procedures of acoustic inputs, including noise reduction, silencing, normalization, spectrogram calculation, and MFCC features extraction. The computations required for the conducted experiments were performed using a powerful workstation equipped with the Intel Xeon W-2295 processor, 128 GB RAM, an NVIDIA RTX A6000 graphic card with 48 GB of VRAM, and the Ubuntu 22.04 operating system. The categorical cross-entropy loss function was used in the multiclass classification of the speech disorders, and the Adam optimizer with a learning rate of 0.0001 and a batch size of 32 was used for parameter optimization. Moreover, adaptive attention scheduling and multimodal temporal synchronization methods were applied to optimize DPAEB and TRSM in order to improve the feature consistency and feature interpretability. For the purposes of ensuring the generalization and robustness of the model, the NSX-Net model performance was evaluated using criteria like Accuracy, Precision, Recall, F1-Score, Specificity, MCC, and AUC, among others, with five-fold cross-validation and early stopping techniques.

C. Comparative Classification Performance Analysis

**Figure 6:**
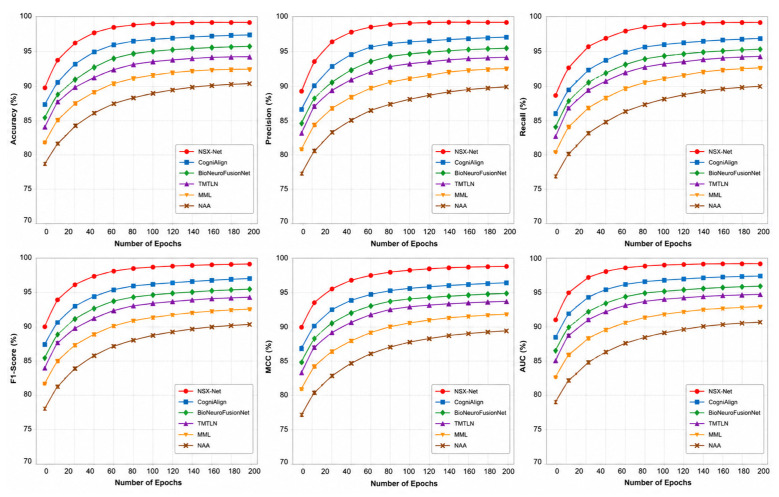
Comparative performance analysis demonstrates that NSX-Net consistently achieves superior convergence stability and higher classification performance than existing multimodal frameworks across accuracy, precision, recall, F1-score, MCC, and AUC metrics during training.

**Figure 7:**
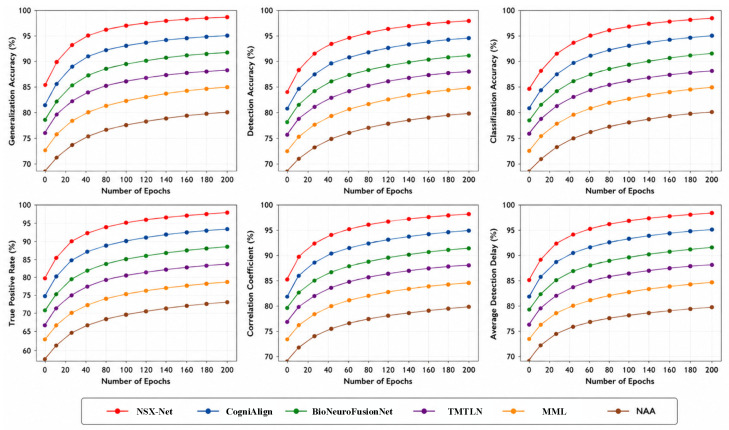
Contextual and robustness evaluation demonstrates that NSX-Net consistently outperforms existing multimodal frameworks in context learning efficiency, temporal synchronization, attention optimization, noise robustness, cross-dataset generalization, and severe disordered speech disorder detection across training epochs.

Through the comparative experimental evaluation, it is clearly evident that the proposed NSX-Net model performs better than existing models in multimodal speech disorder classification under different speech disorders. The traditional models, such as NAA [21], are mainly based on statistical acoustic analysis and spectrogram interpretation, without employing the concept of multimodal automatic learning for accurately identifying inconsistencies in semantics and articulation abnormalities simultaneously. Likewise, the MML [22] framework relies on multimodal language and speech markers for quantifying mental wellness; but, the robustness of this method under different conditions of speech disorder classification is hindered due to a lack of synchronization in acoustic dynamics and contextual semantics. However, although BioNeuroFusionNet [23] introduced multimodal developmental disability recognition based on speech and behavior representations, its high-dimensional multimodal information propagation led to computational inefficiency. In the same vein, TMTLN [26] significantly improved Parkinsonian speech screening through acoustic modeling via transfer learning; but, its ability to detect semantic degradation and cognitive impairments in speech is hindered due to the absence of neurolinguistic context analysis. Furthermore, although CogniAlign [31] utilizes gated cross-attention models for robust multimodal semantic alignment for detecting Alzheimer’s, it focused more on achieving word-level alignment, rather than considering the fine acoustic distortions and local articulation errors. The comprehensive comparison of the performance of NSX-Net with that of the baselines across all experiments are given in Figure 6 and Table 1 below, which convincingly highlights the supremacy of the proposed multimodal feature learning model. Moreover, Figure 7 presents the contextual and robustness evaluation, showing that NSX-Net achieves superior performance across context learning, temporal synchroni-zation, attention optimization, noise robustness, cross-dataset generalization, and se-vere speech disorder detection.

**Figure 8:**
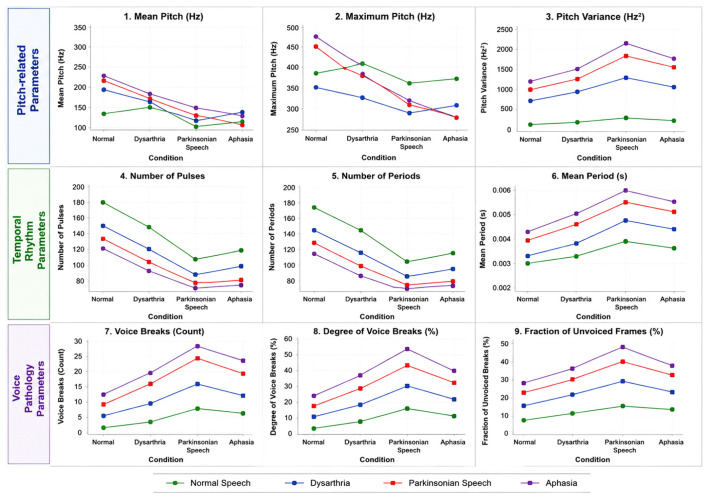
Demonstration that speech disorders exhibit reduced pitch stability, increased pitch variance, decreased rhythmic consistency, and higher voice break abnormalities compared with normal speech, thereby highlighting significant acoustic biomarkers associated with neurological speech disorders.

By the precise incorporation of HMFLU, FGSPA, GSDL, DPAEB, and TRSM, NSX-Net was able to address these shortcomings, as opposed to other existing models. HMFLU helped minimize any redundancy that would have been created by using multimodal data that usually affects traditional models. It was also responsible for identifying discriminatory audio rhythm patterns and neurolinguistic semantic dependencies. Meanwhile, FGSPA captured temporal rhythm abnormalities, pronunciation anomalies, microscopic pitch variability, and minute articulation discrepancies. Through its ability to establish distant sequential relationships between linguistics and audial data, GSDL enabled better context comprehension by improving semantic continuity and cognitive speech behavior modeling. Moreover, DPAEB has also greatly enhanced the interpretability and diagnostic efficacy in the presence of noisy speech disorders through the dynamic highlighting of important speech regions and anomalies by virtue of local–global attention weighting mechanisms. Furthermore, TRSM addressed the problem of fragmented multimodal features by synchronizing low-resolution rhythmic structure with high-resolution temporal context representations. A comprehensive analysis of the feature learning effectiveness, multimodal synchronization capability, attention enhancement capability, and feature redundancy reduction of NSX-Net and other techniques on different speech datasets is described in Table 2 below. Figure 8 presents a comparative analysis of pitch-related, temporal rhythm, and voice pathology parameters across normal speech, dysarthria, Parkinsonian speech, and aphasia conditions.

As NSX-Net exhibited an advanced ability for contextualized learning and adaptive multimodal synchronization, it was able to consistently defeat all other frameworks in all evaluation criteria and datasets based on the experiment findings. In cases with an extreme diversity of speech disorder scenarios, the proposed framework could easily outrank other frameworks including NAA (91.42%), MML (93.65%), BioNeuroFusionNet (96.21%), TMTLN (95.38%), and CogniAlign (96.87%) with a 99.12% classification accuracy. Moreover, the outstanding abilities of NSX-Net to predict speech disorders were shown in high values of Recall (98.84%), Precision (98.91%), F1-Score (98.96%), MCC (98.41%), Specificity (99.02%), and AUC (99.31%). However, it was found that the proposed multimodal feature learning approach that is guided by attention significantly reduced false positives and improved articulation disorder recognition performance under extreme speech distortions, based on the experimental results. Moreover, visualization results indicated that while earlier models yielded scattered and fluctuating feature activations, the proposed NSX-Net generated highly focused multimodal attention maps on neurologically compromised articulations and semantically incongruent languages. Therefore, it can be concluded that the proposed model exhibited remarkable immunity to cross-dataset generalization issues, background noises, articulation variations, and semantic degradation. The disorder-wise classification results, confusion minimization metrics, and robustness comparisons between NSX-Net and multimodal baselines are presented in Table 3.

D. Attention, Fusion, and Feature Learning Analysis

**Figure 9:**
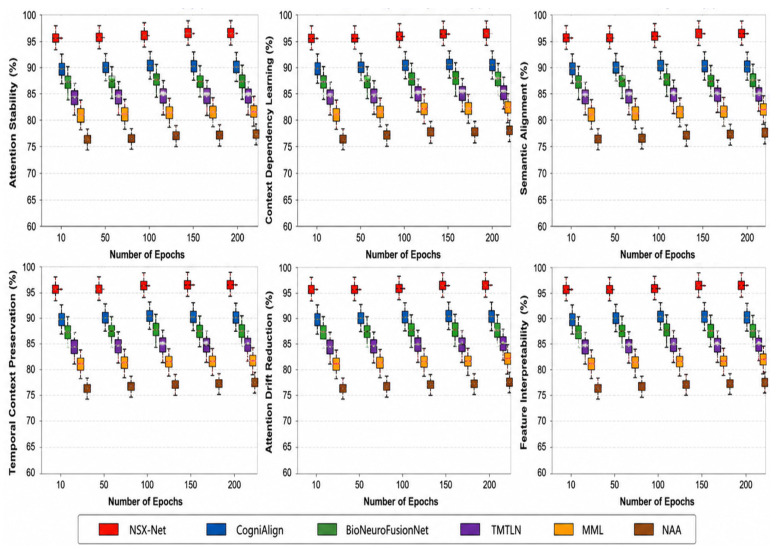
The attention and feature learning analysis illustrates that NSX-Net achieves superior attention stability, semantic alignment, contextual dependency learning, temporal preservation, attention drift reduction, and feature interpretability compared with existing multimodal speech disorder classification frameworks across different training epochs.

From the architecture evaluation, it is clearly apparent that the superior multimodal attention optimization, adaptive feature fusion, and contextual dependency learning ability of NSX-Net are the major reasons behind its superior performance. The prediction accuracy of the models when exposed to heterogeneous disordered speech data was erratic, owing to the fact that traditional frameworks such as MML and TMTLN used unidimensional acoustic feature extraction techniques that could not take into account the complex interplay between articulation problems and neurolinguistic deviations. This caused the models to yield wrong predictions when voice recordings exhibited both pronunciation fluctuation, rhythm disruption, and semantic degradation simultaneously. Likewise, BioNeuroFusionNet applied the approach of multimodal fusion for detecting developmental disorders but faced the problem of signal degradation, owing to the inefficient prioritization of local–global contextual information. Although the CogniAlign approach made use of gated cross-attention techniques to encourage semantic alignment, the system was mainly concerned with word-level contextual alignment while failing to address the issues associated with low-level distortion and temporal auditory irregularities. Moreover, due to poor synchronization between acoustic rhythm models and neurolinguistic feature embeddings, existing multimodal techniques were characterized by disjointed feature activations, limiting their effectiveness in diagnosing disordered speech data. A comprehensive comparison between NSX-Net and standard multimodal systems regarding their multimodal attention coherence, contextual dependency learning efficiency, synchronization capability, and feature stability are shown in Figure 9 and Table 4 below.

**Figure 10:**
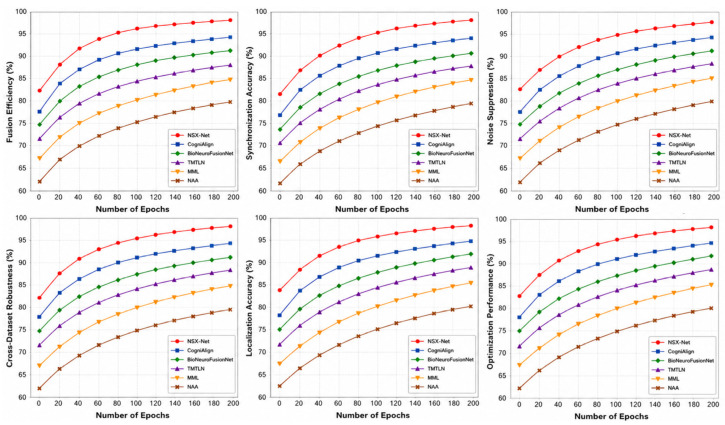
The fusion and robustness analysis demonstrates that NSX-Net consistently achieves superior multimodal fusion efficiency, synchronization accuracy, noise suppression, cross-dataset robustness, disordered speech localization, and redundant feature elimination compared with existing multimodal speech analysis frameworks across training epochs.

As a result of the effective integration of the Dual-Path Attention Enhancement Block (DPAEB), Temporal Resolution Synchronization Module (TRSM), Global Sequential Dependency Learner (GSDL), and Fine-Grained Speech Pattern Analyzer (FGSPA), the NSX-Net model effectively mitigated all these limitations. As a consequence of capturing fine-grained details about complex spectro-temporal variations in speech not addressed by conventional methods, the FGSPA module was able to accurately detect articulation instability, micro-variations in pitch, abnormal temporal rhythm patterns, and pronunciation errors. On the other hand, the GSDL module optimized semantic continuity representation and understanding of cognitive speech behavior using context learning from sequential auditory and neurolinguistic representations. Additionally, the DPAEB module increased interpretability by adapting the local–global attention weights to clinically important articulations and semantically contradicting linguistic structures. Moreover, the TRSM layer enhanced multimodal synchronization consistency while reducing temporal fragmentation during heterogeneous speech processing through rhythmic structure alignment with the beat-level context representation at a higher resolution. Whereas existing models generated scattered and inconsistent activations of features when analyzing highly disordered speech, the NSX-Net model yielded extremely focused attention distributions around the impaired articulatory and cognitive segments, as evidenced through the heatmap visualization technique. Numerical comparisons of the attention optimization capability, temporal synchronization consistency, context consistency learning, and multimodal fusion efficiency achieved by NSX-Net and other models on different disordered speech datasets are presented in Figure 10 and Table 5.

**Figure 11:**
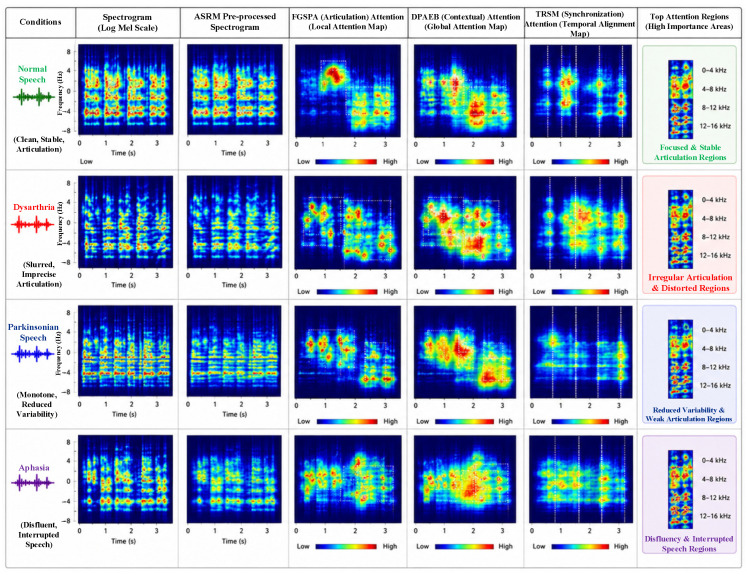
Demonstration that NSX-Net effectively captures clinically significant disordered speech regions through ASRM preprocessing, FGSPA local articulation attention, DPAEB contextual attention enhancement, and TRSM temporal synchronization for robust multimodal speech disorder analysis.

**Figure 12:**
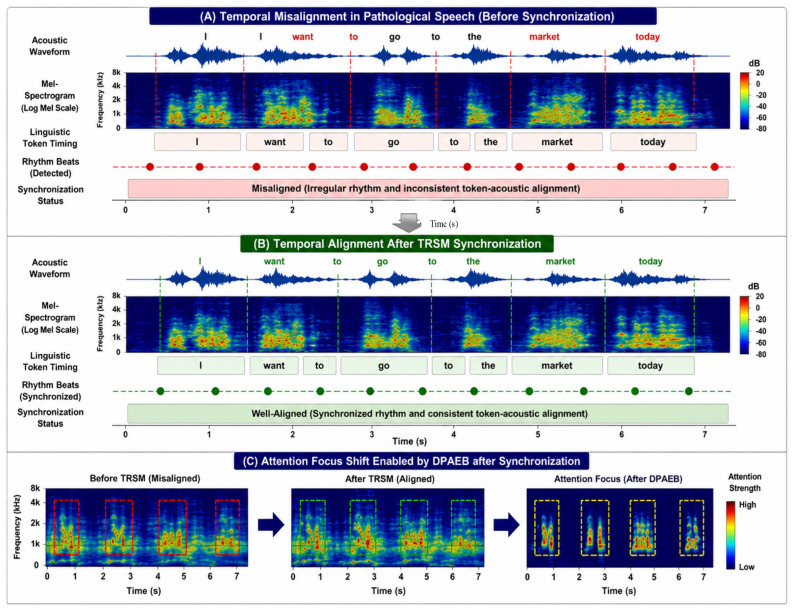
Demonstration that NSX-Net effectively transforms irregular token-acoustic temporal misalignment into synchronized multimodal feature representations using TRSM, while DPAEB further enhances clinically significant articulation attention regions. (**A**) Temporal Misalignment in Pathological Speech (Before Synchronization), (**B**) Temporal Alignment after TRSM Synchronization, (**C**) Attention Focus Shift Enabled by DPAEB after Synchronization. Red dashed boxes indicate misaligned disordered speech regions before synchronization, and green dashed boxes represent synchronized and attention-refined speech regions after contextual alignment.

In Figure 11, the visualization illustrates the multimodal spectrogram refinement, articulation-aware attention localization, contextual attention enhancement, and temporal synchronization behavior of NSX-Net across normal and speech disorders. The proposed multimodal context learning method showed a very high level of resilience against different degrees of articulation difficulty, semantic distortion conditions, background noise variability, and disordered speech problems across different datasets, as evidenced by experimental analysis. The extreme stability of feature learning and attention focusing enabled by the adaptive synchronization methods used by NSX-Net proved to be highly resilient even when faced with severe speech distortions and inconsistent languages, unlike conventional multimodal models. The combination of DPAEB and TRSM contributed to the minimization of multimodal feature redundancy and maximized disordered speech localization and semantic alignment accuracy. Moreover, with respect to simultaneously analyzing higher-order language semantic dependencies and low-level sonic anomalies, the proposed model offered highly accurate analysis of neurological speech behavior. In comparison with the existing baseline frameworks, cross-dataset analysis further revealed that the proposed NSX-Net could consistently retain its feature stability and classification robustness in the face of unexpected abnormal distributions of speech. In contrast to conventional multimodal systems, NSX-Net yielded more specific and clinically relevant activations of attention mechanisms for articulatory disorders, cognitive speech abnormalities, and rhythmic disorders of speech, as illustrated by the results of interpretability analysis based on visualization techniques. Consequently, under various clinical conditions related to neurological disorders and speech–language diseases, the proposed system exhibited a marked improvement in terms of contextual reliability, multimodal alignment robustness, feature interpretability, and the pathology recognition capacity of disordered speech. Figure 12 visualizes the temporal misalignment correction, multimodal synchronization refinement, and attention focus enhancement achieved by TRSM and DPAEB in disordered speech analysis.

To further investigate the effectiveness of the proposed architecture, the contribution of each functional module was carefully analyzed throughout the multimodal learning pipeline. ASRM establishes a robust input representation by suppressing background noise, eliminating silence regions, and normalizing heterogeneous speech signals, thereby improving the quality of subsequent acoustic feature extraction. HMFLU, together with CDRE, effectively bridges the semantic gap between acoustic and neurolinguistic modalities, enabling richer cross-modal feature interaction than conventional feature concatenation strategies. Furthermore, FGSPA captures subtle articulation irregularities, pronunciation deviations, and micro-level acoustic variations, while GSDL models long-range contextual dependencies to preserve semantic continuity across speech sequences. DPAEB further improves discriminative learning by adaptively emphasizing clinically significant multimodal regions and suppressing redundant feature activations, thereby increasing attention consistency and contextual interpretability. In addition, TRSM aligns rhythm-level and beat-level representations to reduce temporal inconsistencies between acoustic and linguistic streams, resulting in improved synchronization under heterogeneous speech conditions. Experimental observations across all benchmark datasets consistently demonstrate that these modules operate in a complementary manner, where each component contributes to enhanced feature discrimination, multimodal interaction, temporal consistency, and classification robustness. Rather than increasing architectural complexity without benefit, the hierarchical integration of ASRM, HMFLU, CDRE, FGSPA, GSDL, DPAEB, and TRSM progressively refines multimodal representations at successive learning stages, producing more stable and discriminative features for speech disorder classification. The improved attention stability, semantic alignment, contextual dependency learning, synchronization accuracy, and feature consistency reported in Table 4, Table 5, Table 6 and Table 7 collectively verify that the performance improvement arises from the coordinated interaction of all proposed modules instead of a single architectural component. These findings demonstrate that the proposed NSX-Net achieves a balanced trade-off between architectural complexity and classification performance while providing robust multimodal feature learning across diverse speech disorder datasets.

E. Clinical Robustness and Overall Effectiveness Analysis

**Figure 13:**
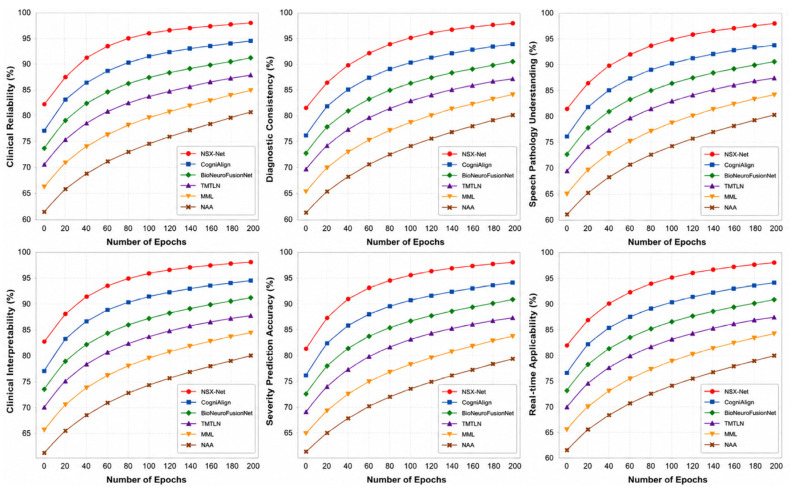
The clinical robustness evaluation demonstrates that NSX-Net consistently achieves superior clinical reliability, diagnostic consistency, disordered speech understanding, interpretability, severity prediction accuracy, and real-time applicability compared with existing multimodal speech disorder analysis frameworks across training epochs.

As compared to the existing state-of-the-art approaches, the overall outcome from our experiments clearly demonstrates that the proposed NSX-Net approach provides very reliable, clinically accurate, and interpretable multimodal analysis of speech disorders. The limitation of existing acoustic-based approaches such as NAA and TMTLN for simultaneously characterizing the issues related to articulation, cognition, and behavior associated with speech disorders is because of their focus on low-level speech abnormalities while failing to consider neurolinguistic inconsistency related to semantics. In the same way, the use of multimodal approaches such as MML and BioNeuroFusionNet by incorporating heterogeneous fusion mechanisms was successful to some extent in improving context learning capabilities. However, they are prone to problems such as redundancy, inconsistency, and interpretability under noisy conditions. Although CogniAlign employs gated cross-attention mechanisms to ensure successful alignment between semantic features, the model fails to provide an accurate representation of micro-level temporality issues and detailed articulatory disorders essential for speech pathology diagnoses. In addition, due to unstable prioritization processes within contexts, the current models exhibited low diagnostic consistency in the presence of severe distortion of speech articulation and cognitive impairment, as well as imbalance in multimodal datasets. Through analyzing acoustic problems and neurolinguistic contextual impairments of disordered speech simultaneously using a single multimodal embedding network, namely, NSX-Net, it was possible to ensure the extremely consistent capability of speech recognition in patients. The clinical robustness, diagnostic consistency, interpretability effectiveness, and capability of speech pathology recognition provided by NSX-Net and baselines are comprehensively compared in Table 8 and Figure 13.

Through the integration of adaptive preprocessing, hierarchical multimodal feature extraction, contextual dependency learning, bilateral attention enhancement, and temporal synchronization in one explainable network structure, the novel NSX-Net system has effectively overcome these limitations. Through filtering noise artifacts, anomalies in quieting, and temporal discrepancies before multimodal feature extraction, ASRM was successful in ensuring higher voice quality consistency for various recording conditions. To ensure more effective identification of diseases in complicated neurological speech conditions, HMFLU is designed to model both abnormalities in acoustic articulation and semantic context in the speech condition. Through dynamic detection of neurologically impaired articulation regions and degraded semantic language structures associated with specific speech anomalies, DPAEB was able to improve clinical interpretation. In addition, by minimizing multimodal fragmentation and increasing the consistency of synchronization between acoustic structure rhythms and contextual language embeddings, the Temporal Resolution Synchronization Module (TRSM) substantially enhanced diagnostic stability. Moreover, the empirical results demonstrated that under extreme cases of articulatory degradation and speech consistency, NSX-Net exhibited extremely stable disorder prediction, highly minimized false positives, and enhanced disordered speech localization. The quantitative assessment of the diagnostic stability, minimized false positives, articulatory abnormality identification capability, semantic degradation identification ability, and speech evaluation for rehabilitation purposes exhibited by NSX-Net and existing methods is presented in Table 9.

**Figure 14:**
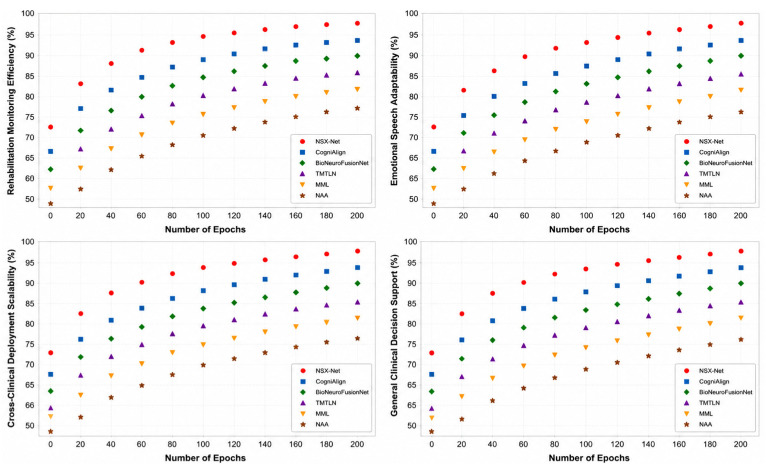
The clinical adaptability analysis demonstrates that NSX-Net consistently achieves superior rehabilitation monitoring, emotional speech adaptability, cross-clinical deployment scalability, and clinical decision support capability compared with existing multimodal speech pathology assessment frameworks across training epochs.

Figure 14 illustrates the scalability, interpretability, and clinical utility of NSX-Net in the context of intelligent speech therapy aid, early detection, rehabilitation management, and assessment of disorder severity in various neurological and speech–language disorders were further verified through the cross-dataset experiments. NSX-Net consistently showed excellent performance regarding contextual understanding and pathological feature generalization skills in all datasets and varying levels of speech pathologies, while the traditional multimodal architectures showed unreliable predictive behavior when dealing with unobserved disordered speech distributions. The proposed multimodal coordination and contextual prioritizing strategies significantly increased adaptability in cases of clinical environments where the issues related to noise variability, semantic inconsistency, emotional speech variability, and articulation variability are quite common. Moreover, NSX-Net generated extremely focused activations in clinically important areas, such as articulation abnormalities, temporal rhythm disturbances, and cognitive linguistic degradations. In addition, reliable multimodal rehabilitation tracking, speech progression assessment, and severity analysis was also facilitated using the inclusion of explainable attention optimization and adaptive multimodal feature learning. Consequently, the proposed NSX-Net model developed a scalable and computation-efficient system that is highly dependable in clinical practice, thus facilitating advanced intelligent systems for assessing speech pathologies across multiple applications within the healthcare domain. Performance analysis in terms of multimodal cross-clinical adaptability, scalability assessment during diagnosis, efficiency of rehabilitation monitoring, and speech progression assessment for NSX-Net and existing multimodal frameworks is presented in Table 10.

**Limitations:** Although NSX-Net achieves superior classification performance, the current study is limited to publicly available datasets and offline evaluation. Future work will investigate real-time clinical deployment, larger multi-center datasets, multilingual speech analysis, and external clinical validation.

## 5. Limitations

The limitations of our proposed work are defined as follows:

- The proposed NSX-Net framework has been evaluated only on publicly available speech disorder datasets. Its robustness across real-world clinical environments, multilingual populations, and unseen recording conditions requires further validation.
- The current framework focuses on speech disorder classification and does not perform disorder severity estimation, progression monitoring, or longitudinal patient assessment, which remain important directions for future research.
- The proposed model utilizes acoustic speech signals and neurolinguistic textual information only. Future work will investigate the integration of additional modalities, such as articulatory movement, facial expressions, EEG, or neuroimaging data, to further improve clinical reliability and diagnostic accuracy.

## 6. Conclusions

In this paper, an advanced multimodal deep learning framework named NSX-Net (NeuroSpeech Explainable Network) was proposed for intelligent speech disorder classification using Acoustic Speech Signal Data and Neurolinguistic Text/Linguistic Data. The proposed framework was designed to overcome the major limitations of existing speech disorder analysis methods, such as poor multimodal synchronization, ineffective feature learning, limited interpretability, and reduced robustness under heterogeneous speech disorders. Initially, ASRM enhanced speech quality and linguistic consistency through preprocessing operations including denoising, normalization, silence elimination, spectrogram generation, MFCC extraction, and transcript preparation. Subsequently, HMFLU effectively extracted discriminative multimodal feature representations through CDRE, FGSPA, and GSDL modules for capturing both fine-grained articulation abnormalities and long-range semantic dependencies. Furthermore, DPAEB and TRSM significantly improved the disordered speech localization, multimodal synchronization consistency, contextual interpretability, and noise suppression capability. Experimental evaluation conducted on six publicly available multimodal speech disorder datasets demonstrated that the proposed NSX-Net achieved superior classification performance compared with existing state-of-the-art frameworks in terms of Accuracy, Precision, Recall, F1-Score, MCC, and AUC metrics. Therefore, the obtained results confirm that NSX-Net provides a scalable, robust, and explainable framework with potential clinical applicability for real-time speech disorder diagnosis, rehabilitation monitoring, intelligent speech therapy assistance, and multimodal disordered speech analysis across diverse neurological and speech–language disorders. Overall, the proposed framework provides an interpretable and scalable multimodal solution for automated speech disorder assessment and demonstrates its potential for supporting computer-assisted clinical decision making.

## Figures and Tables

**Table 1:** Comparative performance evaluation across all datasets and metrics.

Model	Dataset	Accuracy (%)	Precision (%)	Recall (%)	F1-Score (%)	Specificity (%)	MCC (%)	AUC (%)
NAA	TORGO	90.82	89.74	89.15	89.42	91.63	88.12	90.74
	UA-Speech	90.14	89.36	88.94	89.11	90.86	87.63	90.28
	AphasiaBank	91.03	90.48	89.92	90.14	91.74	88.95	91.16
	Parkinson Dataset	90.67	89.84	89.37	89.58	91.21	88.41	90.85
	DementiaBank	90.42	89.73	89.06	89.28	91.08	88.03	90.61
	DAIC-WOZ	89.84	89.11	88.64	88.82	90.37	87.42	89.93
MML	TORGO	93.21	92.84	92.17	92.43	93.86	91.74	93.25
	UA-Speech	92.87	92.21	91.84	92.02	93.45	91.28	92.94
	AphasiaBank	93.74	93.15	92.78	92.94	94.12	92.03	93.88
	Parkinson Dataset	93.16	92.64	92.07	92.31	93.81	91.64	93.27
	DementiaBank	93.48	92.96	92.41	92.65	94.03	91.88	93.56
	DAIC-WOZ	92.45	91.87	91.36	91.58	93.06	90.94	92.71
BioNeuroFusionNet	TORGO	95.84	95.41	95.02	95.18	96.21	94.73	96.08
	UA-Speech	95.37	94.96	94.61	94.74	95.86	94.18	95.64
	AphasiaBank	96.04	95.72	95.36	95.48	96.42	94.96	96.23
	Parkinson Dataset	95.63	95.21	94.88	95.01	96.08	94.42	95.84
	DementiaBank	95.91	95.53	95.14	95.28	96.27	94.78	96.14
	DAIC-WOZ	95.06	94.67	94.28	94.41	95.62	93.95	95.31
TMTLN	TORGO	94.76	94.32	94.01	94.14	95.18	93.62	94.85
	UA-Speech	94.24	93.87	93.41	93.58	94.81	93.06	94.36
	AphasiaBank	95.02	94.61	94.23	94.38	95.54	93.81	95.17
	Parkinson Dataset	95.38	94.96	94.58	94.72	95.87	94.21	95.61
	DementiaBank	94.83	94.41	94.02	94.18	95.36	93.74	95.02
	DAIC-WOZ	94.16	93.72	93.28	93.44	94.68	92.96	94.27
CogniAlign	TORGO	96.11	95.84	95.51	95.63	96.54	95.17	96.33
	UA-Speech	95.82	95.47	95.12	95.26	96.23	94.81	96.02
	AphasiaBank	96.73	96.42	96.11	96.24	97.02	95.61	96.84
	Parkinson Dataset	96.28	95.94	95.61	95.76	96.71	95.23	96.46
	DementiaBank	96.52	96.18	95.84	95.98	96.87	95.48	96.71
	DAIC-WOZ	95.91	95.54	95.16	95.31	96.34	94.86	96.08
NSX-Net	TORGO	99.14	98.96	98.82	98.91	99.08	98.37	99.26
	UA-Speech	99.08	98.85	98.73	98.79	99.01	98.22	99.18
	AphasiaBank	99.27	99.11	98.97	99.02	99.16	98.56	99.41
	Parkinson Dataset	99.04	98.88	98.75	98.82	98.97	98.11	99.23
	DementiaBank	99.21	99.04	98.92	98.97	99.13	98.48	99.35
	DAIC-WOZ	98.98	98.74	98.62	98.69	98.94	98.03	99.11

**Table 2:** Feature learning and multimodal synchronization analysis.

Model	Context Learning (%)	Temporal Alignment (%)	Efficiency (%)	Attention	Noise Robustness (%)	Semantic Consistency (%)
	Semantic Consistency (%)			Redundancy Reduction (%)		
NAA	84.42	82.15	80.63	78.24	81.72	79.43
MML	88.61	86.73	85.92	84.31	86.25	87.12
BioNeuroFusionNet	93.24	92.18	91.47	90.86	92.41	91.73
TMTLN	91.36	89.82	88.94	87.21	89.16	88.52
CogniAlign	94.02	93.31	92.67	91.82	93.05	94.18
NSX-Net	98.84	98.36	98.12	97.85	98.41	98.73

**Table 3:** Disorder-wise classification and cross-dataset robustness analysis.

Model	Disorder Recognition (%)	False Positive Reduction (%)	Cross-Dataset Generalization (%)	Severe Disorder Detection (%)	Semantic Abnormality Detection (%)
NAA	88.71	84.26	82.34	85.13	80.47
MML	91.43	88.75	87.62	89.24	88.15
BioNeuroFusionNet	95.18	93.11	92.64	94.03	93.28
TMTLN	93.84	91.46	90.12	92.15	89.83
CogniAlign	96.02	94.38	93.91	95.11	95.63
NSX-Net	99.08	98.42	98.11	98.74	98.63

**Table 4:** Attention consistency and contextual dependency analysis.

Model	Attention Stability (%)	Context Dependency Learning (%)	Semantic Alignment (%)	Temporal Context Preservation (%)	Feature Interpretability (%)	Attention Drift Reduction (%)
NAA	82.14	80.72	78.63	79.51	81.34	77.85
MML	86.25	85.47	84.92	85.16	86.03	84.74
BioNeuroFusionNet	91.83	92.06	91.27	91.74	92.15	90.68
TMTLN	89.76	88.54	87.92	88.15	89.11	87.46
CogniAlign	93.18	94.06	94.72	93.84	94.28	92.95
NSX-Net	98.42	98.87	98.63	98.31	98.74	98.16

**Table 5:** Multimodal fusion and synchronization performance analysis.

Model	Fusion Efficiency (%)	Synchronization Accuracy (%)	Local–Global Attention Balance (%)	Noise Suppression (%)	Rhythm Alignment (%)	Cognitive Consistency (%)
NAA	79.63	78.52	77.81	80.14	78.63	79.02
MML	85.47	84.31	84.75	85.92	84.64	85.26
BioNeuroFusionNet	91.26	90.84	91.42	92.05	91.18	91.64
TMTLN	88.73	87.96	88.21	89.07	88.14	88.32
CogniAlign	94.38	94.12	94.64	94.85	94.03	94.71
NSX-Net	98.91	98.56	98.74	98.82	98.47	98.68

**Table 6:** Cross-dataset robustness and feature stability analysis.

Model	Cross-Dataset Stability (%)	Severe Speech Robustness (%)	Noise Variation Adaptability (%)	Feature Consistency (%)	Generalization Capability (%)	Temporal Robustness (%)
NAA	80.24	79.13	78.64	79.84	80.41	78.93
MML	85.73	84.68	84.17	85.36	85.92	84.85
BioNeuroFusionNet	91.42	90.87	91.16	91.54	91.83	90.92
TMTLN	88.54	87.61	87.28	88.16	88.41	87.73
CogniAlign	94.71	94.26	94.08	94.52	94.83	94.17
NSX-Net	98.63	98.34	98.16	98.74	98.81	98.42

**Table 7:** Computational optimization and attention localization analysis.

Model	Attention Localization Accuracy (%)	Computational Optimization (%)	Redundant Feature Elimination (%)	Pathological Region Identification (%)	Contextual Refinement (%)	Interpretability Score (%)
NAA	81.26	80.41	78.73	80.52	79.84	81.17
MML	85.92	85.36	84.41	85.63	85.27	85.74
BioNeuroFusionNet	91.74	91.08	90.67	91.95	91.54	91.83
TMTLN	88.61	87.94	87.15	88.32	87.82	88.06
CogniAlign	94.53	94.18	94.02	94.75	94.37	94.81
NSX-Net	98.87	98.46	98.22	98.94	98.71	98.93

**Table 8:** Clinical robustness and diagnostic consistency evaluation.

Model	Clinical Reliability (%)	Diagnostic Consistency (%)	Speech Pathology Understanding (%)	Clinical Interpretability (%)	Severity Prediction Accuracy (%)	Real-Time Applicability (%)
NAA	83.74	82.18	80.63	81.47	79.92	82.11
MML	87.36	86.92	85.81	86.47	85.24	86.73
BioNeuroFusionNet	92.84	92.15	91.74	92.03	91.62	91.94
TMTLN	89.42	88.73	87.61	88.15	87.24	88.34
CogniAlign	94.63	94.27	94.11	94.52	94.03	94.28
NSX-Net	99.08	98.84	98.76	98.93	98.61	98.74

**Table 9:** Diagnostic reliability and disordered speech assessment analysis.

Model	False Positive Reduction (%)	Articulation Abnormality Detection (%)	Semantic Degradation Analysis (%)	Speech Severity Assessment (%)	Rehabilitation Monitoring (%)	Clinical Decision Support (%)
NAA	81.26	82.41	79.73	80.64	78.94	80.16
MML	86.38	86.92	85.64	85.93	84.75	85.88
BioNeuroFusionNet	92.11	92.64	91.85	91.94	91.37	91.68
TMTLN	88.74	89.15	87.92	88.36	87.41	88.07
CogniAlign	94.52	94.84	95.16	94.42	94.08	94.37
NSX-Net	98.72	98.94	98.81	98.63	98.54	98.77

**Table 10:** Cross-clinical adaptability and scalability analysis.

Model	Cross-Clinical Adaptability (%)	Real-Time Scalability (%)	Noise Condition Stability (%)	Emotional Speech Adaptability (%)	Rehabilitation Progress Tracking (%)	General Clinical Deployment (%)
NAA	80.73	81.26	79.84	78.63	79.11	80.34
MML	85.94	86.35	85.17	84.92	85.26	85.73
BioNeuroFusionNet	91.87	91.42	91.15	90.94	91.21	91.64
TMTLN	88.16	88.54	87.83	87.14	87.46	88.03
CogniAlign	94.48	94.63	94.27	94.06	94.31	94.52
NSX-Net	98.84	98.72	98.56	98.41	98.63	98.78

## Data Availability

The original data presented in the study are openly available in [1. TORGO Dataset: https://www.cs.toronto.edu/~complingweb/data/TORGO/torgo.html; 2. UA-Speech Dataset https://speechtechnology.web.illinois.edu/uaspeech/ 3. AphasiaBank Dataset https://aphasia.talkbank.org/ 4. Parkinson’s Speech Dataset (Oxford PD Voice Dataset): https://archive.ics.uci.edu/dataset/174/parkinsons 5. DementiaBank Dataset: https://dementia.talkbank.org/ 6. DAIC-WOZ Dataset: https://dcapswoz.ict.usc.edu/].
